# Multiple Non-Destructive Approaches to Analysis of the Early Silurian Chain Coral *Halysites* from South China

**DOI:** 10.3390/life14081014

**Published:** 2024-08-15

**Authors:** Xinyi Ren, Yazhou Hu, Peiyu Liu, Yue Liang, Feiyang Chen, Hao Qiu, Luke C. Strotz, Kun Liang, Zhifei Zhang

**Affiliations:** 1 State Key Laboratory of Continental Dynamics, Shaanxi Key Laboratory of Early Life and Environments, Department of Geology, Northwest University, Xi’an 710069, China; 201810275@stumail.nwu.edu.cn (X.R.); yue.liang@nwu.edu.cn (Y.L.); lukestrotz@nwu.edu.cn (L.C.S.); elizf@nwu.edu.cn (Z.Z.); 2 Key Laboratory of Deep Petroleum Intelligent Exploration and Development, Institute of Geology and Geophysics, Chinese Academy of Sciences, Beijing 100029, China; liupeiyu@mail.iggcas.ac.cn; 3 School of Resources and Geosciences, China University of Mining and Technology, Xuzhou 221116, China; feiyang.chen@cumt.edu.cn; 4 College of Architecture and Geomatics Engineering, Shanxi Datong University, Datong 037003, China; qiuhao@sxdtdx.edu.cn; 5 Biodiversity Institute and Department of Ecology & Evolutionary Biology, University of Kansas, Lawrence, KS 66045, USA; 6 State Key Laboratory of Palaeobiology and Petroleum Stratigraphy, Nanjing Institute of Geology and Palaeontology, Chinese Academy of Sciences, Nanjing 210008, China; kliang@nigpas.ac.cn

**Keywords:** Tabulata corals, *Halysites*, micro-XRF, micro-CT

## Abstract

Cnidarians are among the most important diploblastic organisms, elucidating many of the early stages of Metazoan evolution. However, Cnidarian fossils from Cambrian deposits have been rarely documented, mainly due to difficulties in identifying early Cnidarian representatives. *Halysites*, a tabulate coral from Silurian reef systems, serves as a crucial taxon for interpreting Cambrian cnidarians. Traditionally, the biological characteristics of *Halysites* have been analyzed using methods limited by pretreatment requirements (destructive testing) and the chamber size capacity of relevant analytical instruments. These constraints often lead to irreversible information loss and inadequate data extraction. This means that, to date, there has been no high-resolution three-dimensional mineralization analysis of *Halysites*. This study aims to introduce novel, non-destructive techniques to analyze the internal structure and chemical composition of *Halysites*. Furthermore, it seeks to elucidate the relationship between coral organisms and biomineralization in reef settings and to compare Silurian Tabulata with putative Cambrian cnidarians. Techniques such as micro-X-ray fluorescence spectrometry (micro-XRF), micro-X-ray computed tomography (micro-CT), and scanning electron microscopy (SEM) were employed in this research. With the help of high-resolution micro-CT scanning, we identify the growth pattern of *Halysites*, showing both lateral and vertical development. The lateral multiple-branching growth pattern of *Halysites* corals is first established herein. The flaggy corallite at the initial stage of branching is also observed. The micro-XRF mapping results reveal the occurrence of septa spines for *Halysites*, a trait previously thought rare or absent. Additionally, the ratio of coral volume to the surrounding rock was assessed, revealing that *Halysites* reefs were relatively sparse (volume ratio = ~30%). The cavities between *Halysites* likely provided more space for other organisms (e.g., rugose corals and bryozoans) when compared to other coral reef types. Additionally, we provide a comparative analysis of post-Cambrian colonial calcareous skeletons, offering insights into the structural features and growth patterns of early skeletal metazoans across the Ediacaran–Cambrian boundary.

## 1. Introduction

Cnidarians are considered one of the most basal metazoan groups, representative of the earliest stages of Eumetazoan evolution. However, soft-bodied diploblastic fossils are infrequently found in pre-Cambrian and Cambrian deposits [1,2,3,4], meaning the earliest stages of cnidarian evolution have been difficult to ascertain. Corals, which represent significant reef-builders throughout the fossil record, secrete a mineralized exoskeleton similar to those produced by stromatoporoids and archaeocyathids, forming calcareous skeletons or spicules to reinforce their body structure [5,6,7]. One of the more common diploblastic skeletal clades found in the fossil record is the Tabulata, which were prevalent during the Paleozoic era, from the early Ordovician to the Permian, and are distributed across the globe [4,7,8,9,10,11,12,13,14].

*Halysites* is an important genus of Tabulata, having previously been used as a model taxon for interpreting the affinities of Cambrian metazoan taxa in carbonate deposits [1,15,16,17]. Characteristic of Tabulata, *Halysites* exhibit developed tabulae with weakly or slightly developed septa [17,18,19]. Previous researchers have examined the morphology, classification, and evolutionary significance of *Halysites* using thin sections and have documented its distribution and stratigraphic occurrences [16,20,21,22,23,24]. However, more recent research on *Halysites* has focused less on its biology, with a large portion of recent studies concentrating on its utility in oil and gas exploration [7,8,9,10,12,13,18,22,25]. Traditional methods of analyzing *Halysites* have relied on hand samples, polished slabs, and thin sections, techniques that are usually destructive [20,26,27], resulting in the loss of potential details and an inability to conduct multi-dimensional analyses.

**Figure 1:**
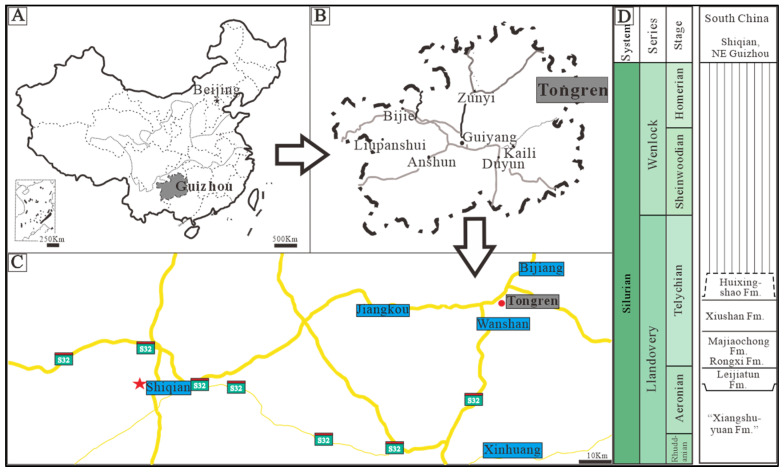
Location of the Shiqian section (red star) and its associated stratigraphic section. (**A**) the location of the study area in China (gray area); (**B**) the location of the study area in Guizhou Province (marked with a gray rectangle); (**C**) a route map of the sampling area (red-star marking the studied area) (modified from [28,29,30]); (**D**) stratigraphic column for the upper part of the Xiangshuyuan Formation in the early Silurian (modified from [17]).

To extract more information, advanced near-non-destructive technologies and methods, such as scanning electron microscopy (SEM), micro-X-ray fluorescence spectrometry (micro-XRF) and micro-X-ray computed tomography (micro-CT) were employed on well-preserved early Silurian examples of *Halysites* from the upper Xiangshuyuan Formation, Guizhou Province of South China (Figure 1), to investigate their 2D and 3D morphological characteristics. Additionally, this study utilizes Silurian Tabulata as a model to identify potential Cnidarians in Cambrian small shelly fossil faunas from comparable carbonate deposits.

## 2. Materials and Methods

More than 100 *Halysites catenularius* (Linnaeus, 1767) [31] specimens were collected from dark gray or grayish white limestones of the upper part of the Xiangshuyuan Formation, early middle Silurian, Shiqian County, Tongren, Guizhou of China (Figure 1). Four larger samples were selected for this research; two of them were selected for non-destructive instrumental analysis, and the other two were used to create thin sections. The first sample contains 172 coral individuals, and the second sample contains 179 coral individuals. All specimens are deposited in the collections of the Department of Geology of Northwest University, Xi’an, China. Fossils were examined under the binocular Zeiss Zoom Stereo microscope (Zeiss, Oberkochen, Germany) and were photographed using a stereo photographic Zeiss Smart Zoom 5 (Zeiss, Oberkochen, Germany). Three-dimensional non-destructive internal micro-structure was obtained using a Zeiss Xradia Versa 520 micro-CT (micro-X-ray computed tomography) (Zeiss, Oberkochen, Germany) [32,33], with a spatial resolution of 0.7 µm and 70 nm voxel imaging. The micro-CT data were reconstructed using the Dragonfly software 3.1. The surface structure of the corals was assessed using scanning electron microscopy (Quanta 400 FEG; FEI, Hillsbora, America). To obtain the chemical composition of *Halysites* specimens, micro-X-ray fluorescence spectrometry (micro-XRF) was used (Brucker, Karlsruhe, Germany), including mapping elemental analysis, which was used to compare the elemental differences between the fossil and surrounding rock, as well as the different parts of the fossils themselves [34,35,36,37,38,39,40,41,42,43]. The proportion of area occupied by *Halysites* was determined using micro-XRF images and using the ImageJ software 1.48 [31,44,45,46].

## 3. Results

### 3.1. Two-Dimensional Morphology and Mineralized Structure of Halysites

**Figure 2:**
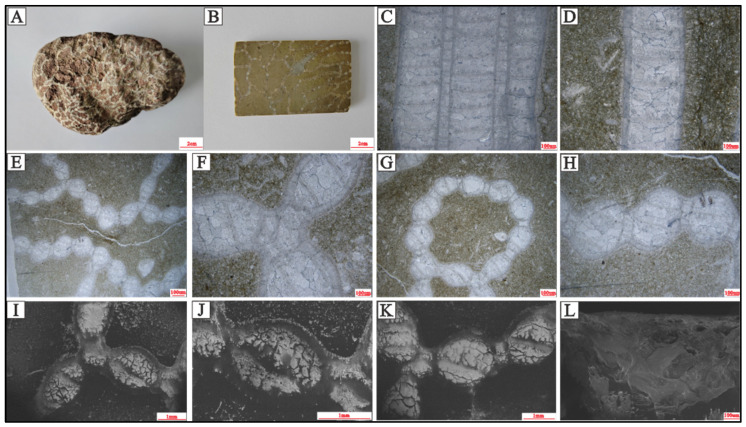
*Halysites* from the upper part of the Xiangshuyuan formation at Shiqian County, Guizhou, South China. The corallites are closely arranged, and corallite walls are relatively thick. (**A**,**B**) external view and photo of polished slab, (**C**,**D**) longitudinal sections under the polarized light, (**E**–**H**) transverse sections under the polarized light, and (**I**–**L**) transverse section images taken with SEM. *Halysites* corallites are connected in a chain-like pattern, with no fixed angle or number. Some form lacunae with up to 10 corallites, while others may extend further away. Tabulae are well-developed, flat, or slightly concave, and tubules share thick walls with corallites. The corallite’s cavity fills have structures similar to the septa.

*Halysites catenularius* (Linnaeus, 1767) develops polygonal lacunae (Figure 2E), and rank junctions occur at tubules (Figure 2C). Additionally, corallites are subelliptical (Figure 2E–K). *Halysites* colonies are always arranged as chains composed of many individuals and are all linked by coenenchymal tubules (small tubes). Colonies often produce new individuals on both sides of the coral’s long axis. The individual corallites are long and thin tubes, round or oval in shape, and the corallite walls (Figure 2A,B,F,J) consist of two layers: the outer wall, which is in contact with the external environment, and an inner wall, which surrounds the tubule chamber. The crystallization of the corallite walls compared to the coral cavity is notably different (Figure 2J). The fossilized parts of *Halysites* are gray-white, the matrix is dark gray, and *Halysites* individuals are connected by tubules, forming variously sized meshes or longitudinal and transverse tabula (Figure 2B).

**Figure 3:**
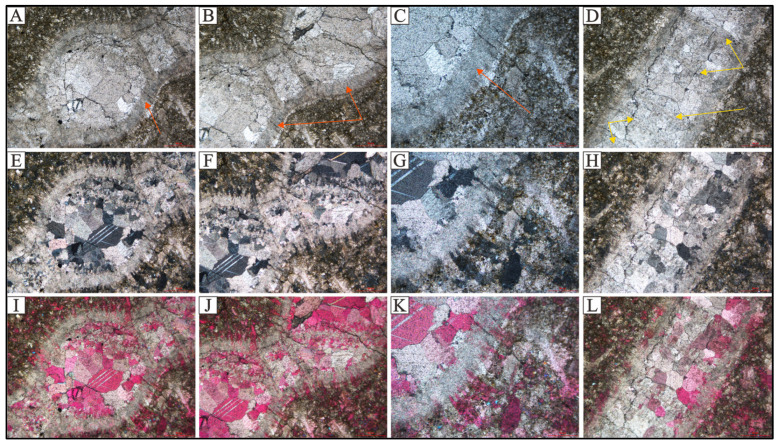
Photomicrographs of *Halysites* captured under polarized light (**A**–**D**) and polarized microscope images (**E**–**L**) using different test plates to distinguish the structure of each part. (**A**,**E**,**I**) represent individual corallites (red arrows) with tubules on two sides, and the matrix is brown, while the corallite wall is dark gray, and the fill consists of coarse-grained crystals. The tubules wall and corallites wall are composed of fine mineral particles, and their fills have a similar composition. (**B**,**F**,**J**) show corallites are connected by tubules, and tubules are rectangle-shaped. The tubule wall is thinner than the corallite wall. (**C**,**G**,**K**) show the boundary of corallites and the surrounding matrix. The fill consists of coarse calcite particles, the corallite wall is composed of very fine particles, and the matrix has a mixture of minerals and particle sizes. (**D**,**H**,**L**) show the composition and particle size for tabulas (yellow arrows), fills, and matrices. The tabulas consist of fine particles, similar to the corallite walls (red arrows), and corallite walls are thicker than the tabulas.

The shape of the tubules is variable (quadrilateral or triangular, predominantly quadrilateral), and they are filled with calcite material similar to the calcite that fills the main chambers. Numerous irregular fractures are observed in the longitudinal section (Figure 2C,D). The tabulae are fully developed, straight, or slightly concave in the middle part of the colony. The fill between the tabulae also has numerous cracks, but no cracks develop on the tabula, and the cracks in the surrounding matrix do not cross them. The tubules are multilayered longitudinally, with the longitudinal single tube not possessing a uniform width and occasionally tapering abruptly upward. No septal spines were observed under SEM or polarizing microscopy (Figure 2 and Figure 3).

### 3.2. Three-Dimensional Morphology of Halysites

**Figure 4:**
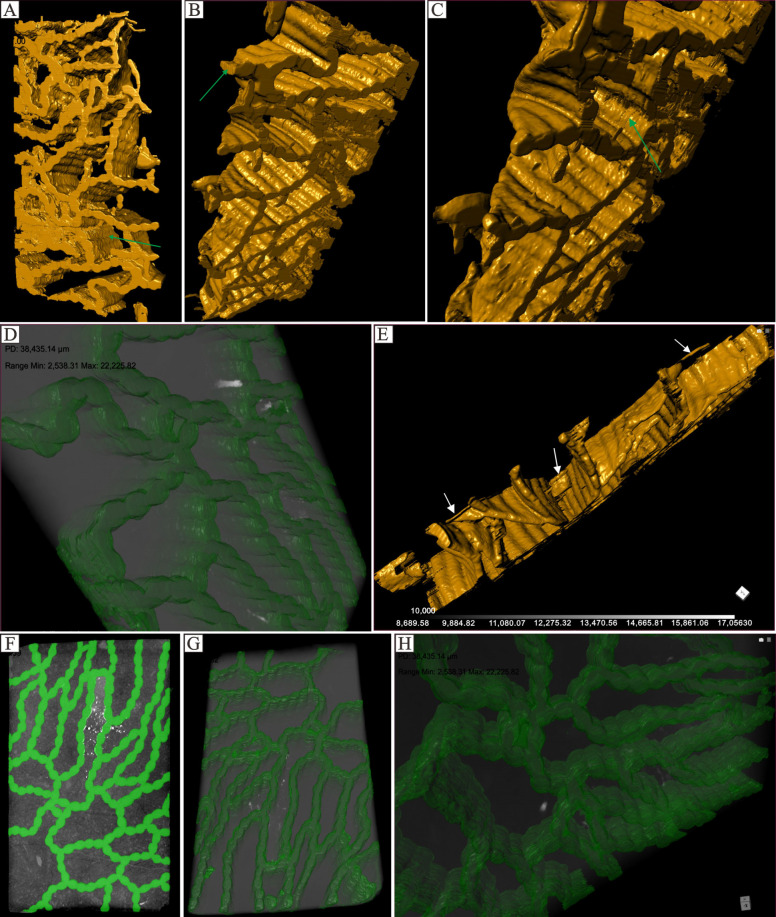
Three-dimensional reconstruction of *Halysites* based on micro-CT. The resolution of the first set of micro-CT images is 38.22 μm/pixel (images with yellow false color), and for the second set (green false color), the resolution is 41.13 μm/pixel. (**A**–**C**,**E**) display corals (yellow) extracted from the matrix; (**D**,**F**–**H**) depict corals (light gray) against a gray background; (**A**–**C**) illustrate the shape of the coral when connected with others, and tabula that are oriented in a longitudinal direction can be seen (green arrow); (**E**) newly forming branches at the top of the specimen (white arrow).

**Figure 5:**
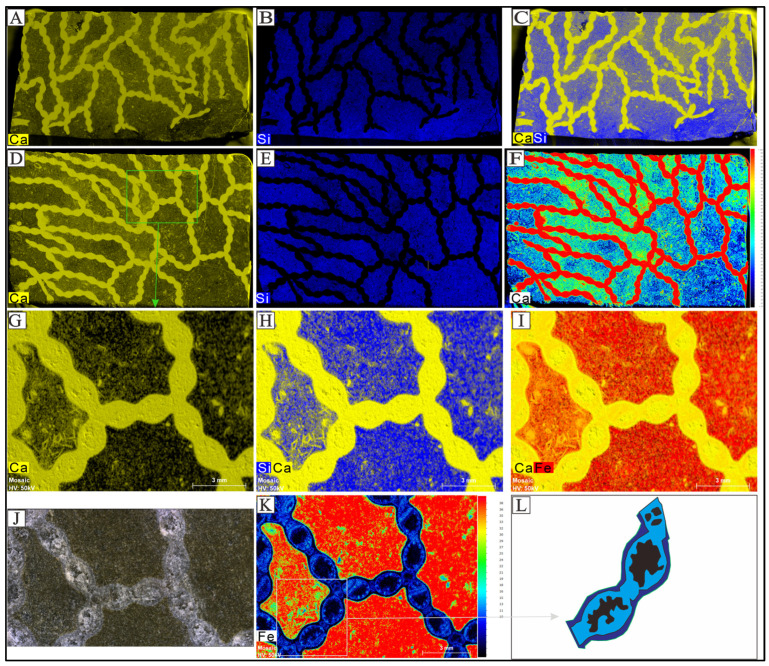
Elemental mapping of *Halysites* skeleton and surrounding sedimentary matrix using micro-XRF imaging (Ca—calcium, Fe—iron, Si—Silicon). (**A**,**D**,**G**) distribution maps of Ca, G located in the green rectangle of D; (**B**,**E**) distribution maps of Si; (**C**,**H**) coupled distribution maps of Si–Ca; (**J**) polished section of *Halysites*; (**I**) coupled distribution maps of Fe–Ca; (**F**) heatmap showing the distribution of Ca abundance; (**K**) heatmap showing the distribution of Fe abundance; (**L**) a schematic drawing of the coral calyx. The content of the light gray box in (**K**) is interpreted to represent the remains of coral polyps and septal spines. Purple represents the coral tube wall, blue represents the coral calyx, and black represents the coral cavities.

**Figure 6:**
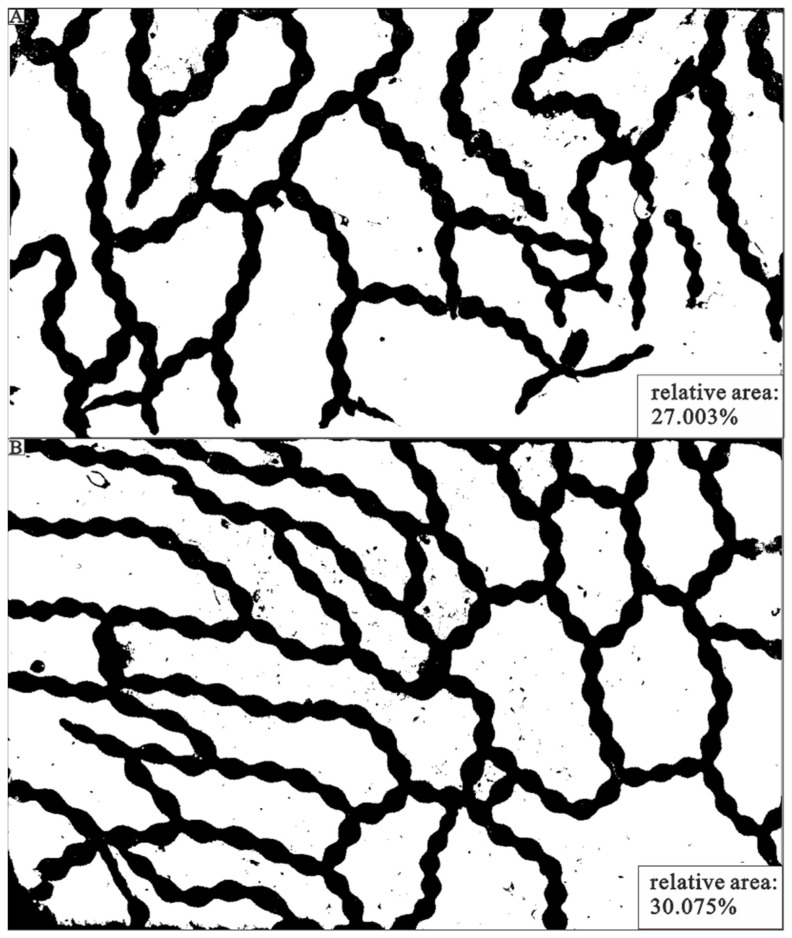
The relative area of *Halysites* versus the surrounding matrix. The black chains represent the coral skeleton, and the white represents the matrix component. In the matrix, there are some scattered broken coral fragments. (**A**) The area occupied by *Halysites* takes up 27.003%, and in (**B**) 30.075%.

**Figure 7:**
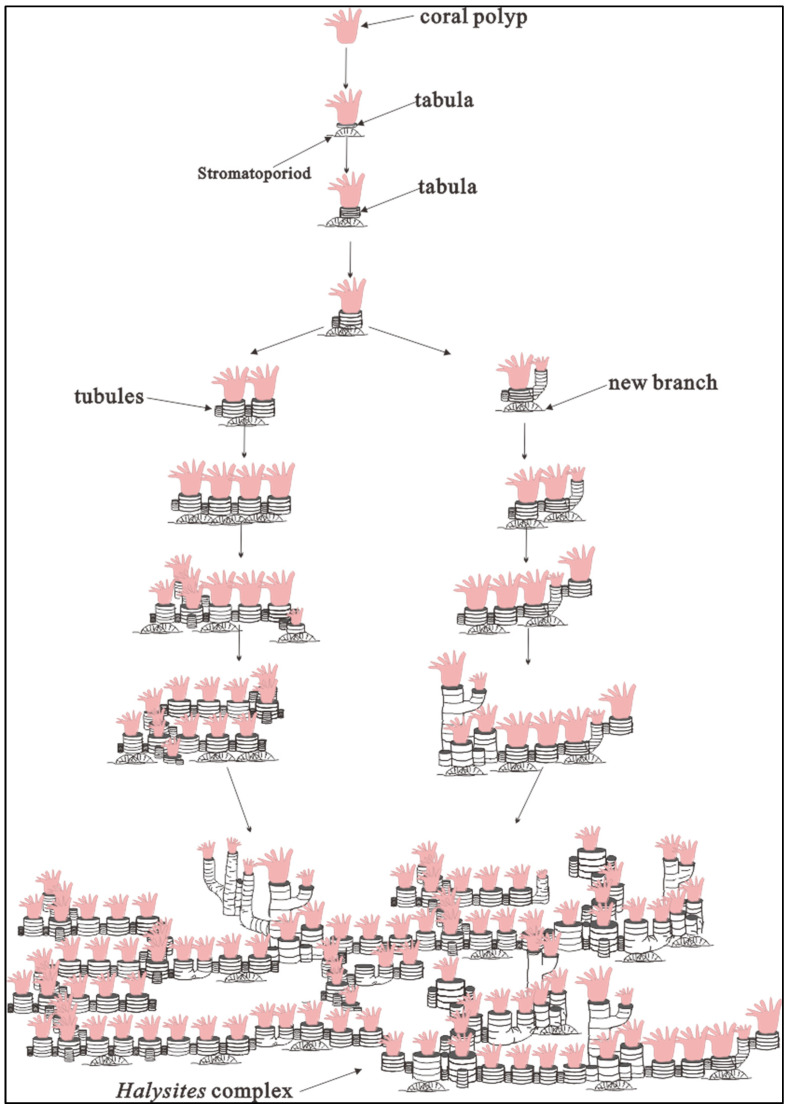
A reconstruction to illustrate the *Halysites* development process [47]. At the beginning, the coral larvae mature and begin to secrete calcite to produce the corallite exoskeleton, forming the first tabula of the *Halysites* colony. Coral polyps continuously produce layered horizontal tabulae, which ultimately lift polyps away from the original substrate. Meanwhile, with the help of tubules, the newly formed ranks branch in multiple directions, resulting in the complex structure typical of *Halysites* reefs.

*Halysites* form irregular lateral ranks or lacunae, which are organized into multiple longitudinal columns and are closely connected laterally to create a fence-like structure. The examined *Halysites* fossils are well preserved, consisting of complete three-dimensional forms with no signs of fragmentation or fracture. Due to the similarity between coral skeletons and associated infilling material, *Halysites*’ internal structures, such as connecting tubules, cannot be identified. The *Halysites* chain extends horizontally and connects to surrounding *Halysites*, exhibiting a horizontal layered growth pattern (Figure 3, Figure 4, Figure 5, Figure 6 and Figure 7). Some specimens grow in a straight line, while others bend or grow in layers. At the top, they continue to grow in the vertical direction. Each group of monomers can connect to two or even four adjacent groups of *Halysites*. *Halysites* develop vertically in an upward direction and reproduce at certain heights. Specifically, the coral’s tube splits around the middle of the coralla. Micro-CT shows that as they grow upwards, the corallum becomes larger compared to the base, and the area at the highest point is also wider, as indicated by the white arrow (Figure 4E). *Halysites* forms in chains but still possess the short, columnar, longitudinally stacked shape that is characteristic of the Tabulata. Consequently, the mesh morphology of *Halysites* coralla is diverse and flexible.

### 3.3. The Elemental Analysis of Halysites

Elemental distribution mapping was performed using micro-XRF to obtain semi-quantitative elemental distributions (Figure 5). Results of these analyses show that, although Ca is distributed in both fossil and matrix regions, the region occupied by the fossil has a stronger calcium signal compared to the matrix (Figure 5A,D,G). The Ca signal (represented in yellow false color) is markedly stronger in the coral structures, while Si and Fe signals (represented in blue and red false colors, respectively) were weak in the regions occupied by the coral and much stronger in the matrix (Figure 5A,C). Analysis of the mapping tests indicates that the septal spines are well preserved, and it can be seen that inside each coral individual, there is a calyx-like structure, but this feature is difficult to distinguish in hand specimens or under polarized light. Micro-XRF mapping of Fe and Ca highlights the position and distribution of the septal spines. Septal spines are very irregular in shape, and some are divided into two or three parts. The septal spines are short and thin, and some corallites do not have septal spines. The size and number of septal spines varies (4–12 septal spines). The minimum number is due to internal space splitting, resulting in 4–6 septal spines. In addition, there are also structures similar to septal spines in the connecting tubules that are sometimes connected to each other, with 2–3 possible radiating arrangements (Figure 5K,L). The length of the septal spines ranges from 0.094 mm to 0.450 mm.

### 3.4. Halysites Reef Volume Calculation

For volume calculations, more than 100 samples from similar preservation conditions were collected, and two larger slabs were selected, both containing multiple individuals connected in chains, with the chosen slabs best fully representing the preservation and morphology of *Halysites*. ImageJ software was used to determine the proportion of fossils to the total rock area. The connecting tubules and coralla were distinguished in the software by coloring them black, with the surrounding rock represented by white. In the fossil areas, some white debris was mixed in, representing secondary sediment infilling. Similarly, black fragments were found in the surrounding rock, indicating that fossils had weathered or become fragmented and mixed with the rock. The software calculated the relative area occupied by fossil material on the first block to be 27.003%. The relative area of the second block is calculated to be 30.075% (Figure 6).

## 4. Discussion

### 4.1. New Insights into 2D and 3D Morphological Reconstruction of Halysites

Halysitid corals first appeared in the early late Ordovician and disappeared in the end of the Silurian [31,48]. Although the strange chain-shaped halysitid corals are abundant [31,33,49], the 2D and 3D morphological reconstructions have not been well established. The development of the growth pattern of halysitid colonies has been the focus of previous studies, which were solely based on thin sections or polished slabs, resulting in many insights into halysitid corals’ skeleton development [50]. No details about the development of the whole halysitids have been established. Although the CT scanning method has been used in halysitids studies, the resolution is low (with 125 μm/pixel in [51]), making it hard to make precise reconstructions of the whole halysitids coral. Meanwhile, the CT scanning by [51] focuses on the growth pattern but without only vertical development information, leaving no details on how halysitids coral growth in horizontally. The non-destructive method in 2D and 3D views (micro-CT and micro-XRF) by hand samples offers several advantages than solely based on thin sections or polished slabs (e.g., [31,51,52,53]). The high resolution herein (with 38.22 μm/pixel and 41.13 μm/pixel) reveals the high contrast between coral skeletons (coral cavity fills) and the surrounding matrix, providing an excellent view of the growth pattern of *Halysites* that is better than previous research. As mentioned before, branching growth patterns are always expected near the lateral edge of the whole chain in corals (Figure 4E). The single colony would be branched into two or three colonies, with the branching angle ranging from 60° to 120°. Meanwhile, there are also some branches occurring with only one colony, possibly representing the dying colony (Figure 6B). Considering the complete morphology revealed by micro-CT scanning, we proposed that branching exists in the coral colony skeleton structure and that branching is a growth pattern for reef expansion. Based on the high-resolution micro-CT scanning results, we made a reconstruction to illustrate the *Halysites* development process (Figure 7). In the beginning, the corallite will inhabit a hard support (e.g., shells or skeletons of other organisms) [5], and then the pre-existing rank will be elongated by the insertion of new coral zooids (Figure 7). After the vertical increase of corallite, followed by a lateral increase by branching the new ranks, it will be linked with old coral zooids by intercorallite dissepimental tissue [53]. With multiple laterally branching methods, a polygonal-shaped *Halysites* coral reef is formed (Figure 2B and Figure 4, Figure 5 and Figure 6).

Although micro-CT reveals coral structures in a high resolution. The contrast between the coral wall or coral original skeleton and the sparry calcite fills is low, resulting in no further detailed analysis by using micro-CT. Micro-XRF offers non-destructive elemental testing and has recently gained popularity in sedimentology, paleontology, and geology [34,35,37,38,54,55]. The fossilized corals studied herein are about 2 mm in diameter of the corallite and under the microscopic XRF field of view, showing high resolution of *Halysites* details (Figure 4 and Figure 5). Micro-XRF analysis indicates the distribution of silica, calcium, and iron, revealing a five-part structure in chain corals (Figure 5K). Specifically, the iron element mapping results of micro-XRF show some irregular tiny structures in the coral zooids that could be the septal spines of *Halysites*. Septal spines in *Halysites* do not always exist [16,19,28,56], and this is particularly true for the observation from polished slabs and thin sections observed by microscope (Figure 2 and Figure 3). Due to the different structures and functions, there must be subtle differences in the composition of the outer epithelial when secreting coral walls and septal spines. However, under the micro-XRF scanning, septal spines are intensely occurring in most *Halysites* coral zooids (Figure 5K,L), implying that there are limitations in traditional microscopic observation technology and micro-XRF could provide high-resolution internal structure analysis of *Halysites* coral zooids. This might be due to the ability of organic matter to adsorb iron elements in sediments and preserve them in the form of complexes that the abnormal iron signal is due to the residual organic carbon in the fossil burial process, which enriches the iron element in the fluid and forms hematite [57]. In other words, the iron signal shows the organic soft-bodied original position and the septal spines might be produced through coral’s secretion and mineralization. Therefore, the internal skeleton structure could be revealed by different elemental comparisons in micro-XRF.

### 4.2. Implications for Halysites Reef Formation

Coral reefs have been regarded as one of the most important examples of ecosystem engineering, creating multiple niches for other organisms [17,19]. Coral reef formation in the Paleozoic is usually primarily a result of biomineralization by the coral themselves, although sometimes aided by other organisms such as stromatoporoids, pelmatozoans, and bacteria [58]. The initial growth of coral reefs always requires the presence of a hard substrate (e.g., shells, skeletons, or lithified rocks [44]). *Halysites* is no exception to this pattern, and the formation of *Halysites* reefs starts with a single corallite. As the colony expands, both vertically and laterally, a complex *Halysites* reef is formed. In our study area, the *Halysites* themselves only take up ~30% of the total available volume, with the cavity between *Halysites* chains filled with soft matrix (Figure 6). However, those cavities are not always filled with soft matrix in other study areas. A recent study of Silurian *Halysites* from the Lower Visby Formation, Gotland, Sweden, reveals that the halysitid colonies there have been used as a hard substrate for rugose corals to stabilize their position in what is a soft sediment environment [26]. The rugose corals also occupy the cavities between halysitid ranks. Compared with tabulate coral reefs constructed by other taxa, *Halysites* reefs seemingly provide relatively more space for other organisms to inhabit.

### 4.3. Implications for Small Shelly Fossils Assessment

Tabulate corals appeared in the middle Ordovician and diversified in the Silurian and Carboniferous periods. Cnidarians have been repeatedly reported from early Cambrian deposits, but no diploblast examples, e.g., tabulata, have been found [1,2,3,49,56]. There are many tubular fossils from Cambrian deposits that remain unclassified, and there may be traces of Tabulata, but evidence is lacking in mudstone or shale [49]. Consequently, it is advantageous to seek out three-dimensional preserved Eumetazoa in carbonates, where such fossils may be preserved in great detail and obtained through acid dissolution. After etching the carbonates, the phosphatized steinkerns are left, resulting in isolated elements of a single taxon that may be interpreted as different species. For example, cylindrical microtubules of likely corals were interpreted as stems and carpal branches of crinoids [59]. Similar microtubes have also just been classified as tubular fossils [60]. More recent work has revealed these tubes should be considered basal cnidaria and were similar to younger coral taxa [61,62,63,64]. These tubular fossils have similar structures to those observed for *Halysites* in our study (Figure 3), including a tectum, inner–outer tectoria, and a core filled with different minerals [65,66,67]. This suggests the possibility that some of these tubular fossils may be the unrecognized tabulata.

## 5. Conclusions

Multiple 2D and 3D nondestructive methods were performed on specimens of *Halysites* for the first time, allowing detailed analysis of *Halysites* morphology with no damage to the actual specimens. With the help of high-resolution micro-CT scanning, we identify a pattern of growth for *Halysites*, with both lateral and vertical development and evidence of multiple branching. The layered corallite at the initial stage of branching is also observed. Elemental distribution analysis of the *Halysites* skeleton, coral domatia, and surrounding matrix allows for clear differentiation of the coral morphology and ultrastructure. Results of micro-XRF mapping reveal the occurrence of large concentrations of septal spines in *Halysites* corallites that were previously thought rare or absent. Additionally, based upon an assessment of the volume occupied by *Halysites* colonies, it seems *Halysites* occupied only approximately 1/3 of the available space, with the cavities between *Halysites* chains providing space for other organisms (e.g., rugose coral and bryozoans). The reconstruction of *Halysites* herein may provide valuable reference data for seeking basal or stem-group cnidarians in Cambrian deposits.

## Data Availability

Data related to this study can be found in Zenodo (10.5281/zenodo.12804814).
